# Single‐Cell Computational Frameworks for Quantifying BET Bromodomain Inhibitor Resistance and Screening Re‐Sensitizer Drugs in Triple‐Negative Breast Cancer

**DOI:** 10.1002/advs.202513246

**Published:** 2026-04-03

**Authors:** Haizhou Liu, Mengqin Yuan, Yini Shang, Jiahao Chen, Fei Hou, Lihong Wang, Wei Jiang

**Affiliations:** ^1^ Fujian Key Laboratory of Tumor Immunotherapy The First Affiliated Hospital Fujian Medical University Fuzhou China; ^2^ National Regional Medical Center Binhai Campus of the First Affiliated Hospital Fujian Medical University Fuzhou China; ^3^ Department of Bioinformatics Fujian Key Laboratory of Medical Bioinformatics School of Medical Technology and Engineering Fujian Medical University Fuzhou China; ^4^ Department of Pathophysiology School of Medicine Southeast University Nanjing China; ^5^ Department of Biomedical Engineering Nanjing University of Aeronautics and Astronautics Nanjing China

**Keywords:** BET bromodomain inhibitor, drug resistance, ferroptosis, scRNA‐seq, triple‐negative breast cancer

## Abstract

Triple‐negative breast cancer (TNBC) is an aggressive subtype characterized by rapid proliferation and a great propensity for metastasis. Therapeutic options for TNBC remain limited due to the absence of targetable hormone receptors. While BET bromodomain inhibitors (BBDIs) exhibit promising anticancer potential, the emergence of drug resistance presents a major challenge. Here, through leveraging single‐cell RNA sequencing (scRNA‐seq) data across continuous states of BBDI treatment in TNBC, this study conducts an extensive investigation into BBDI resistance, and develops two computational frameworks, FR20 and D‐FR20, to quantify BBDI resistance at single‐cell resolution and to screen potential BBDI re‐sensitizer drugs, respectively. The accuracy and scalability of FR20 are confirmed through rigorous evaluation in nine independent datasets. In addition, cellular dynamic changes and ferroptosis inhibition are revealed in the evolution of BBDI resistance. Experimental validation demonstrates that *GPX*4 overexpression significantly reduces drug sensitivity in TNBC cells. Furthermore, in vitro and in vivo experiments validate the ability of the small molecule filgotinib, identified by D‐FR20, to re‐sensitize BBDI and effectively eliminate resistant TNBC cells. Collectively, this study provides two computational frameworks for predicting BBDI resistance and candidate re‐sensitizer, as well as demonstrates the roles of ferroptosis in BBDI resistance, offering a promising avenue for TNBC treatment.

## Graphical abstract

To combat BET inhibitor resistance in triple‐negative breast cancer, we developed two single‐cell computational frameworks. FR20 quantifies resistance by integrating ferroptosis‐related gene signatures, while D‐FR20 screens for potential re‐sensitizers. The efficacy of both algorithms was rigorously validated through multi‐omics analyses and multi‐layered in vitro and in vivo experiments. This integrated strategy provides a precise toolkit for overcoming therapy resistance.

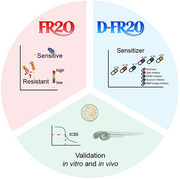

## 1 Introduction

Triple‐negative breast cancer (TNBC), which accounts for approximately 15%–20% of all breast cancer patients, is the most aggressive subtype. Currently, chemotherapy is one of the most common treatments for TNBC [1, 2]. Unfortunately, 60% to 70% of TNBC patients do not exhibit a favorable response to chemotherapy [2]. BET bromodomain inhibitor (BBDI) is regarded as a potentially novel candidate to treat TNBC in preclinical experiments [3]. BBDI could shortcut the communication of BRD4 and transcription of downstream oncogenes, resulting in killing tumor cells [4]. However, while these agents show strong preclinical activity, their clinical translation has been challenging, particularly in solid tumors, as evidenced by limited responses in clinical trials [5]. A key obstacle is the development of intrinsic and acquired resistance, which has been consistently documented in cell lines and xenograft models [5], but remains mechanistically unresolved. Thus, understanding the underlying mechanisms of BBDI resistance and refining therapeutic strategies is helpful to breast cancer treatment.

Another important issue is substantial heterogeneity in TNBC. The genomic diversity and transcriptional heterogeneity between different patients (inter‐tumor) or within the same patient (intra‐tumor) can lead to cells with different drug responses [6, 7]. Yang et al. identified four TNBC subtypes by analyzing 465 patients [8]. The luminal androgen receptor (LAR) subtype, characterized by the up‐regulation of oxidized phosphatidylethanolamines and glutathione metabolism showed higher sensitivity to ferroptosis inducers [8]. In a neoadjuvant chemotherapy‐resistant TNBC patient, there was a coexistence of both sensitive and resistant clones [9]. Furthermore, even within the resistant clones, copy number variations were observed, suggesting different molecular characteristics [9]. The diverse resistance mechanisms pose a significant challenge for cancer treatment [10]. Currently, single‐cell RNA sequencing (scRNA‐seq) technology offers opportunities to explore the heterogeneity of drug response at a single‐cell resolution. Zhang et al. demonstrated that TNBC with high levels of baseline *CXCL*13^+^ T cells had better responses to the combination of paclitaxel and anti‐PD‐L1 atezolizumab by scRNA‐seq analysis of 22 patients [11]. Even within the same cell line, there exist varying drug responses among the cells. For example, a rare pre‐existing cell subpopulation in TNBC cell line with higher expression of *IGFBP*2, was demonstrated greater resistance to afatinib compared to other cells [12]. Thus, quantification of single‐cell drug responses in TNBC can uncover BBDI resistance heterogeneity, providing a roadmap for precision therapies.

In addition, drug combination therapy has proven to be an effective method to overcome drug resistance. For example, afatinib has shown potential in re‐sensitizing MRTX1133‐resistant pancreatic ductal adenocarcinoma cells [13]. Both in vitro and in vivo, lansoprazole has exhibited its capacity to enhance sensitivity to paclitaxel in metastatic melanoma cells [14]. The Connectivity Map (CMAP, https://clue.io) and the Library of Integrated Network‐based Cellular Signatures (LINCS, http://www.lincsproject.org), which compile extensive data on small molecule‐induced transcriptomes, provide valuable resources for predicting potential drugs and drug combinations [10, 15, 16]. Thus, identifying combination therapies offers opportunities for overcoming BBDI resistance.

**FIGURE 1:**
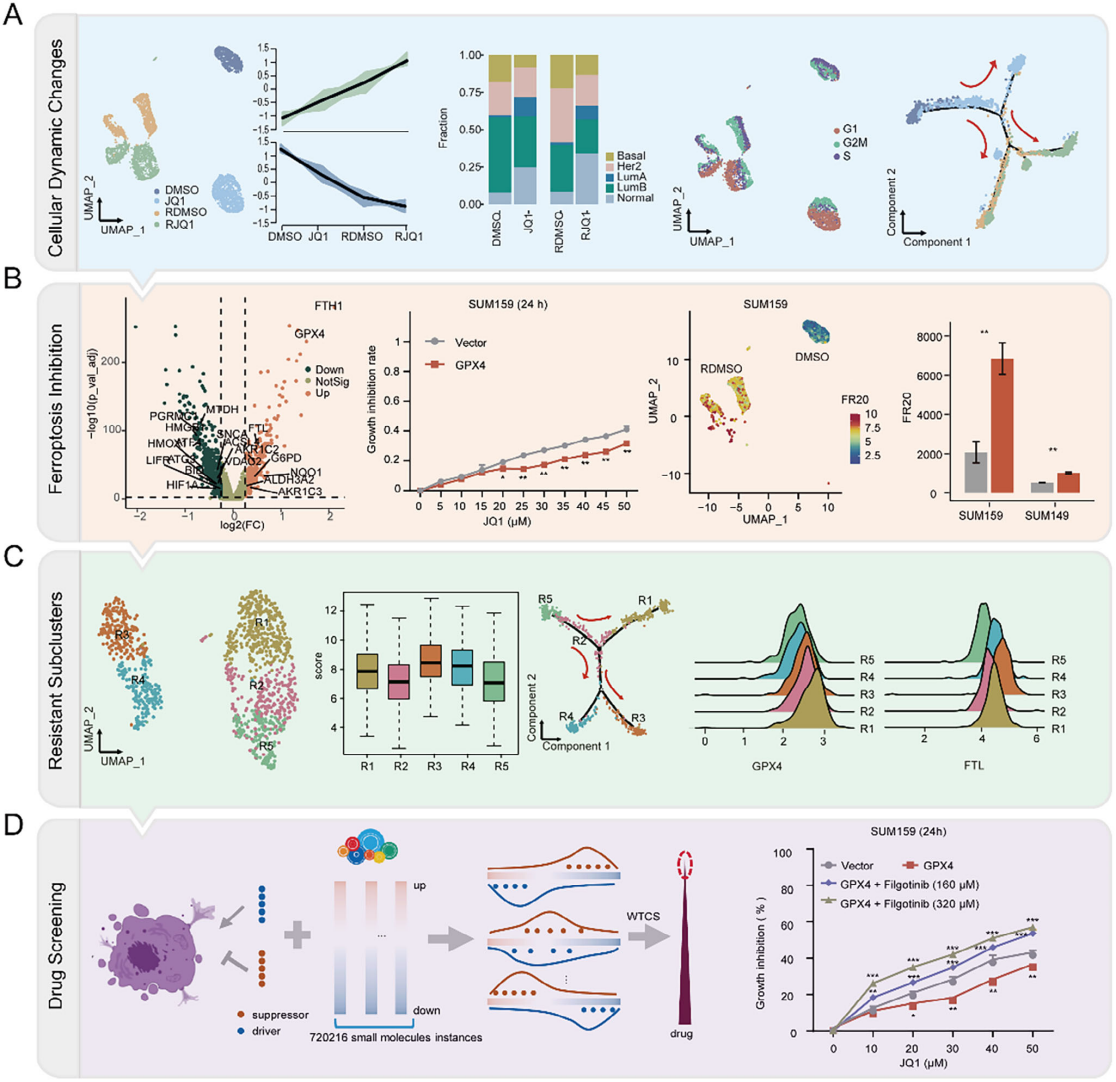
Workflow diagram for this work. (A) Cellular dynamic change analysis during JQ1 treatment and resistance from the aspects of transcriptome, cellular composition, cell cycle, and evolution time. (B) Ferroptosis inhibition in JQ1 resistant cells. (C) Heterogeneity of JQ1 resistant cells. (D) JQ1 re‐sensitizer screening and validation.

Here, to systematically characterize the dynamic changes in BBDI resistance evolution and identify potential drugs reversing resistance, we developed two computational frameworks and performed in vitro and in vivo experimental validation through leveraging the time series scRNA‐seq data from JQ1‐sensitive to resistant of TNBC cells (Figure 1). First, we revealed the cellular dynamic changes during the progression of JQ1 resistance. Next, we discovered and validated the pivotal roles of ferroptosis underlying JQ1 resistance. In addition, we proposed FR20 framework to quantify the level of JQ1 resistance for an individual cell. The accuracy and scalability of FR20 were validated by nine independent multi‐omics datasets. Furthermore, we observed significant heterogeneity in the resistant cells with different drug responses to JQ1. Finally, we proposed D‐FR20 framework to screen JQ1 re‐sensitizer and validate the efficacy of filgotinib. Collectively, our study developed two computational frameworks to predict JQ1 resistance and re‐sensitizer, and illuminated the role of ferroptosis in JQ1 resistance and the ability of filgotinib to overcome drug resistance.

## 2 Results

### 2.1 Cellular Dynamic Changes Under JQ1 Treatment

**FIGURE 2:**
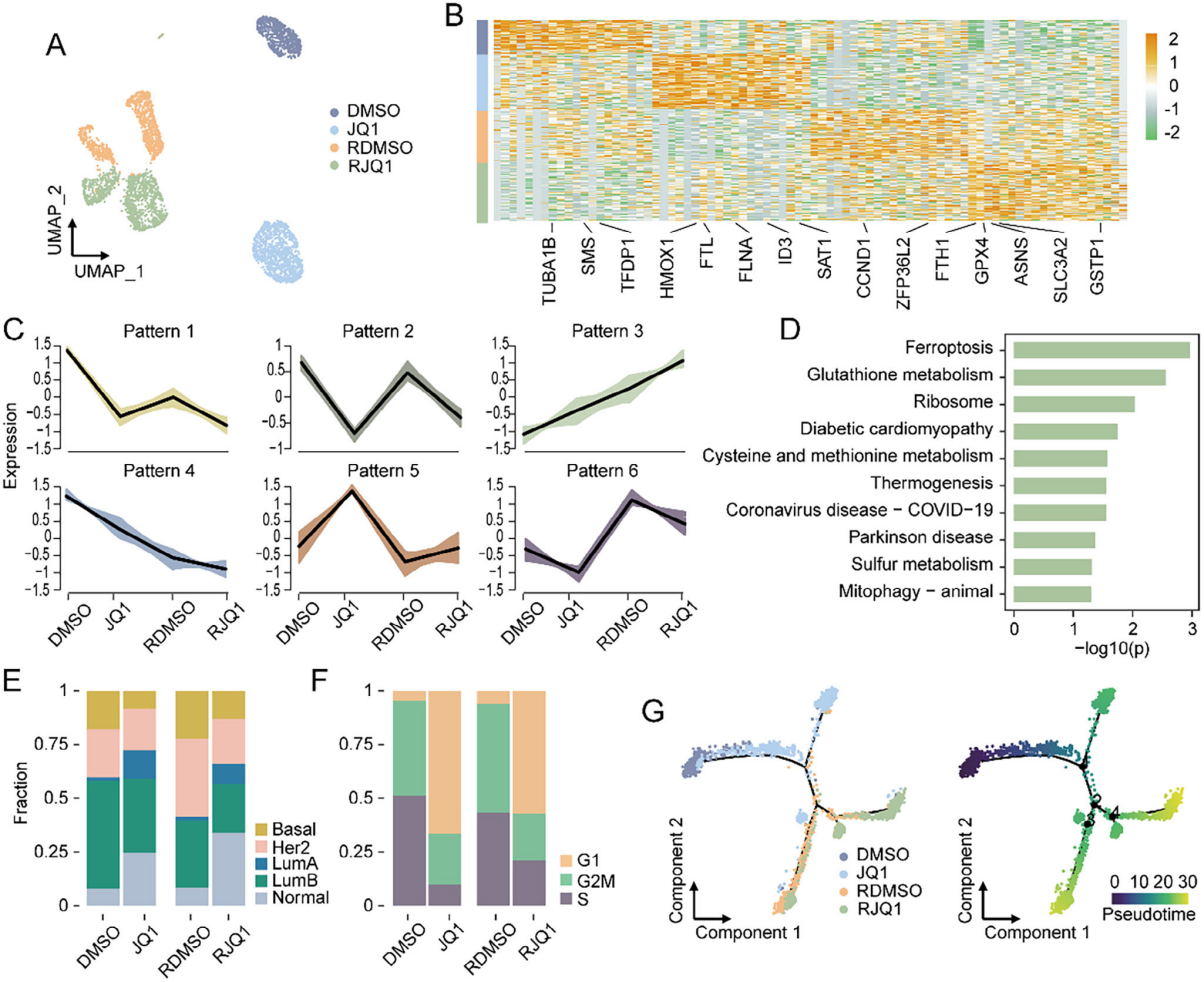
Characterizing cellular dynamic changes during JQ1 treatment and resistance by scRNA‐seq. (A) Uniform manifold approximation and projection (UMAP) visualization of SUM159 cells colored by sensitive cells (DMSO), JQ1‐treated cells (JQ1), resistant cells (RDMSO), and resistant cells retreated with JQ1 (RJQ1). (B) Heatmap of the top 20 markers in each stage. (C) Six dynamic patterns of gene expression. Differentially expressed genes (DEGs) clustered by their expression pattern, only genes with membership > 0.6 were shown. (D) Bar graph of the top 10 KEGG pathways with the smallest *p* values enriched in genes of pattern 3. (E) Cell proportion of different PAM50 subtypes in each stage. (F) Cell proportion of different cell cycle phases in each stage. (G) Lineage trajectory of cells in four stages inferred by Monocle2. Cells are labeled by pseudotime (right).

We comprehensively characterized the dynamic changes during JQ1 treatment and resistance. After filtering low‐quality cells, 751 sensitive (DMSO), 1200 JQ1‐treated (JQ1), 1130 resistant (RDMSO), and 1285 resistant cells retreated with JQ1 (RJQ1), were analyzed. Clustering analysis revealed clear separation and increased heterogeneity, transitioning from one cluster in sensitive cells to two in resistant cells (Figure 2A; Figure S1). Differential expression analysis identified dynamically reprogrammed genes (log_2_ *FC* > 0.25 and *FDR* < 0.01), including cell cycle‐related genes (such as *CCND*1, *FLNA*, *TFDP*1, *TUBA*1*B*, *ZFP*36*L*2, *ID*3, *ASNS*) and ferroptosis‐related genes (such as *FTH*1, *FTL*, *GPX*4, *HMOX*1, *SAT*1, *SLC*3*A*2, *GSTP*1, *SMS*), indicating mechanism of JQ1 action and resistance (Figure 2B; Table S1). Then, mfuzz analysis revealed six dynamic expression patterns, with patterns 3 and 4 showing steady up‐regulation and down‐regulation, respectively (Figure 2C; Table S2). Pattern 3 genes were enriched in ferroptosis‐related pathways, such as “ferroptosis” and “Glutathione metabolism” (Figure 2D), while pattern 4 genes were linked to protein synthesis and degradation, such as “ribosome” and “proteasome” pathways (Figure S2). These results showed dynamic molecular changes from sensitive to resistant states.

We further analyzed phenotypic changes using the PAM50 model [17]. JQ1 treatment reduced the basal subtype proportion and increased the normal‐like subtype, with minimal changes in luminal subtypes (Figure 2E), consistent with findings that basal breast cancer cells are more sensitive to JQ1 than luminal cells [3]. Additionally, JQ1 treatment increased the proportion of cells in the G1 phase (Figure 2F), aligning with its role in cell cycle arrest [18, 19]. Pseudotime analysis revealed a branched progression trajectory from sensitive to resistant states (Figure 2G). These results revealed the characteristics of cellular dynamic changes during JQ1 treatment and resistance.

### 2.2 Involvement of Ferroptosis‐Related Genes in JQ1 Resistance

**FIGURE 3:**
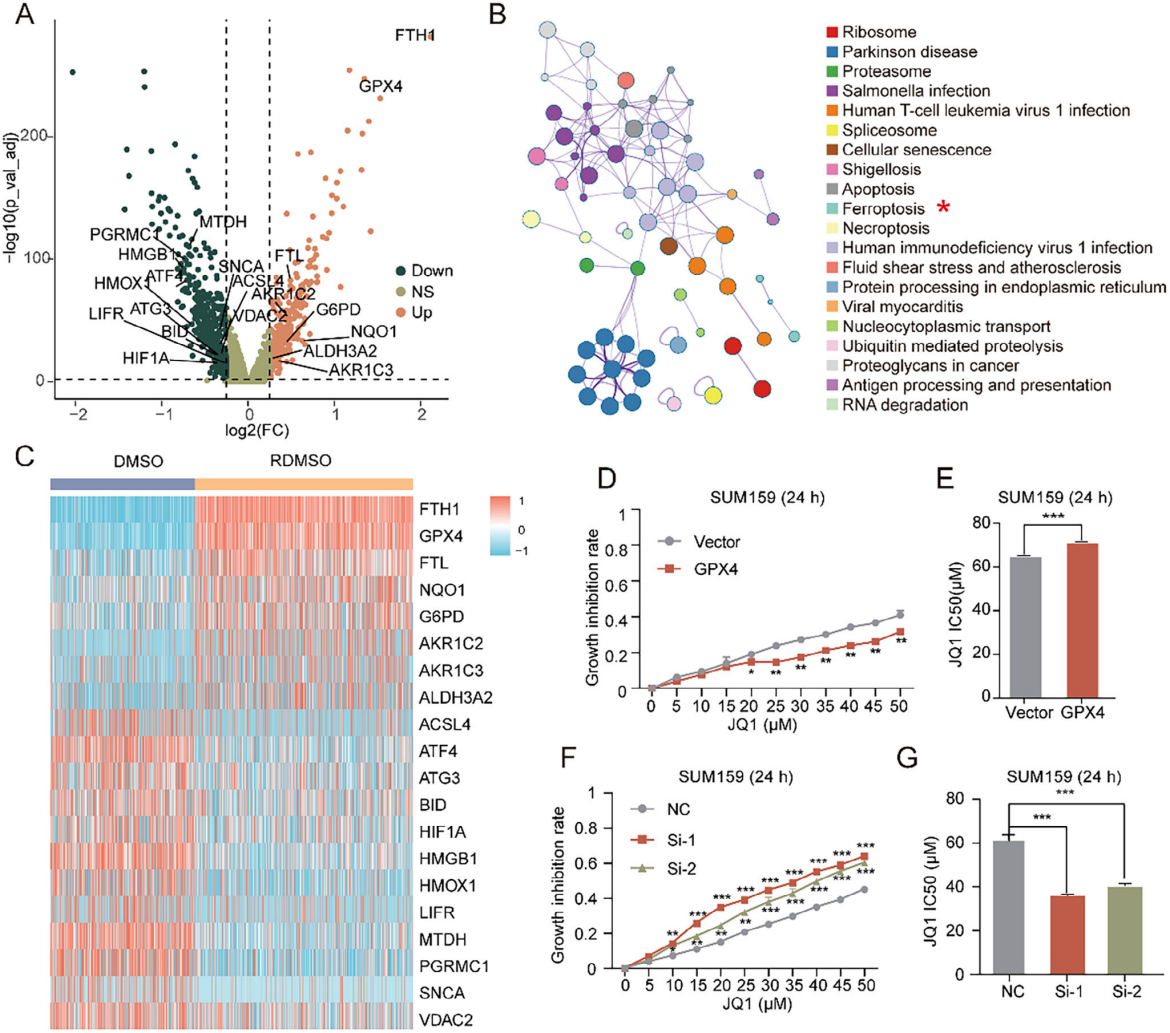
Ferroptosis modulation by differentially expressed genes (DEGs) in JQ1‐resistant cells. The overexpression of *GPX*4 leads to resistance to JQ1. (A) Volcano plot of DEGs between resistant and sensitive cells. The ferroptosis genes are labeled. (B) Enrichment map of KEGG pathways for the DEGs between resistant and sensitive cells. The ferroptosis pathway is highlighted with star. (C) Heatmap of significantly differentially expressed ferroptosis genes in resistant and sensitive cells. Color scale represents normalized expression levels (red: up‐regulated; blue: down‐regulated). (D) CCK8 assay of JQ1 inhibition rate (24 h) in *GPX*4 overexpression group versus Vector control group. (E) IC50 values of JQ1 (24 h) in the *GPX*4 overexpression group versus the Vector control group. (F) CCK8 assay for JQ1 inhibition rate (24 h) in *GPX*4 knockdown group versus Vector control group. (G) IC50 values of JQ1 (24 h) in *GPX*4 knockdown group versus the Vector control group. Data represent mean ± SD, Significance in (D and E) was calculated using the Unpaired‐samples t tests (n = 3). Significance in (F and G) was calculated using the one‐way ANOVA (n = 3). *: *p* < 0.05; **: *p* < 0.01; ***: *p* < 0.001.

To explore JQ1 resistance mechanisms, differential gene expression analysis identified 328 up‐regulated and 633 down‐regulated genes, with ferroptosis suppressors *FTH*1 (log_2_ *FC* = 2.116, *FDR* = 3.519 × 10^−282^) and *GPX*4 (log_2_ *FC* = 1.348, *FDR* = 4.179 × 10^−248^) among the top up‐regulated genes (Figure 3A). Notably, overexpression of *FTH*1 and *GPX*4 confers ferroptosis inhibition by reducing iron accumulation [20] and lipid peroxidation [21], respectively. Moreover, KEGG pathway enrichment analysis for these differentially expressed genes (DEGs) further highlighted the ferroptosis pathway (Figure 3B,C; Table S3), emphasizing its role in JQ1 resistance.

To assess well‐known ferroptosis regulator *GPX*4's role, SUM159 cell lines with *GPX*4 overexpression and knockdown were established (Figure S3A–D, uncropped Western blot membrane: see Supporting Information). The *GPX*4 overexpression decreased JQ1 inhibition rates and increased IC50 values (Figure 3D,E; Figure S3E,F), while *GPX*4 knockdown enhanced JQ1 sensitivity, with higher inhibition rates and lower IC50 values (Figure 3F,G; Figure S3G,H). Taken together, these results underscored the critical role of *GPX*4 in JQ1 resistance and highlighted its potential as a therapeutic target.

### 2.3 FR20 is Capable to Predict Risk of Resistance to JQ1

**FIGURE 4:**
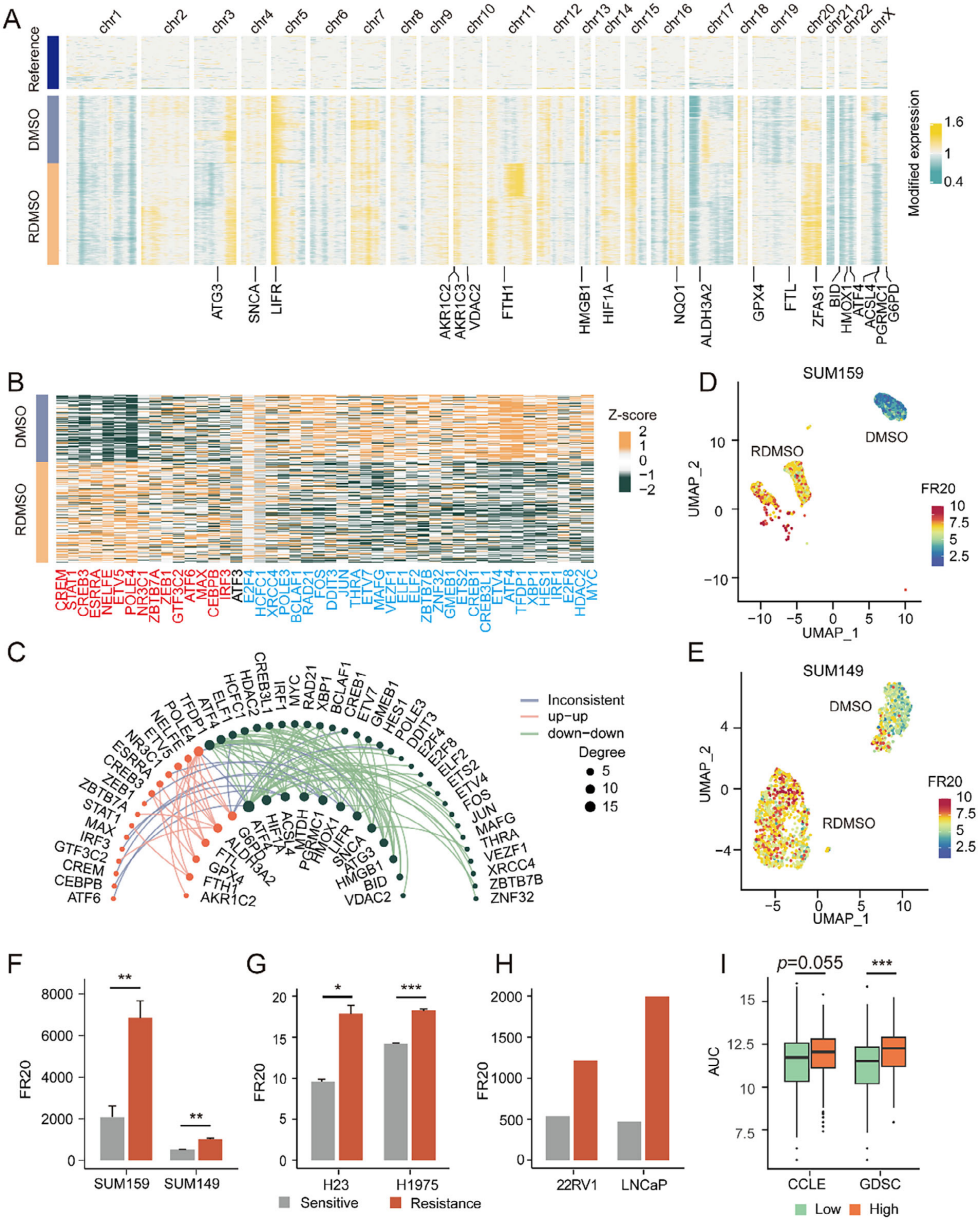
Discovery and validation of FR20. (A) Heatmap of inferred copy number variation (CNV) across normal epithelial (Reference), sensitive, and resistant cells. (B) Heatmap of transcription factors (TF) activities in resistant and sensitive cells. Red (blue) text represents that the activity of TF was significantly up‐regulated (down‐regulated) in resistant cells (two‐sided Wilcoxon Rank‐Sum test *p* < 0.05). Black represents no significant difference. (C) Network of TF and the regulated 18 ferroptosis regulators. The edges represent the regulations between TFs and the ferroptosis regulators. Red (green) nodes represent significantly up‐regulated (down‐regulated) ferroptosis regulators or activated (inactivated) TFs in resistant cells. The red (green) edges represent TF and the regulated gene were consistently up‐regulated (down‐regulated). The gray edges represent inconsistent dysregulation direction. (D, E) UMAP visualization of FR20 for single cells in JQ1 resistant and sensitive cells of SUM159 (D) and SUM149 (E) cell lines. (F–H) Bar plots of FR20 for JQ1 resistant and sensitive samples from GSE131135 (F), GSE164813 (G), and GSE103082 (H). (I) Boxplots of AUC for top and bottom quartile cell lines ranked by FR20 score in GDSC and CCLE, respectively. Significance in (F and G) was calculated using the two‐tailed t‐test. Significance in (I) was calculated using the two‐sided Wilcoxon Rank‐Sum test. ***: *p* < 0.001; **: *p* < 0.01; *: *p* < 0.05.

We conducted further analysis of ferroptosis‐related regulators, revealing distinct expression patterns between suppressors and drivers. Notably, up‐regulated genes showed significant enrichment among ferroptosis suppressors (*p* = 0.0093; Figure S4A), whereas down‐regulated genes were preferentially enriched in ferroptosis drivers (*p* = 0.0429; Figure S4B). These findings suggest an inhibition of ferroptosis in JQ1 resistance. Specifically, 8 ferroptosis suppressor (*FTH*1, *GPX*4, *G*6*PD*, *AKR*1*C*2, *AKR*1*C*3, *ALDH*3*A*2, *NQO*1 and *FTL*) were up‐regulated and 12 ferroptosis drivers (*ACSL*4, *ATF*4, *ATG*3, *BID*, *HIF*1*A*, *HMGB*1, *HMOX*1, *LIFR*, *MTDH*, *PGRMC*1, *SNCA* and *VDAC*2) were down‐regulated. Furthermore, we conducted a systematic analysis of these 20 genes in resistant and sensitive cells. Copy number analysis revealed significant changes (two‐sided Wilcoxon Rank‐Sum test *p* < 0.05) in 17/20 genes in resistant cells (Figure 4A; Figure S4C). Gene regulatory network (GRN) analysis identified 46 TFs regulating 18/20 genes, with 45/46 TFs showing differential activity (two‐sided Wilcoxon Rank‐Sum test *p* < 0.01, Figure 4B; Table S4). In 85/97 (87.6%) pairs, TF dysregulation aligned with gene expression changes (Figure 4C), highlighting transcriptional regulation of ferroptosis‐related genes in JQ1 resistance.

To quantitatively assess the risk of JQ1 resistance, we developed a ferroptosis resistance score FR20 by integrating gene expression of 20 ferroptosis regulators (details in Materials and Methods). In the Discovery Cohort (SUM159 scRNA‐seq), resistant cells had higher FR20 scores (two‐sided Wilcoxon Rank‐Sum test *p* < 2.2 × 10^−^^1^ ^6^, Figure 4D; Figure S4D). Validation across nine independent cohorts (Table 1) confirmed FR20's accuracy (More details in Supporting Information). SUM149 TNBC scRNA‐seq (Validation Cohort I) showed higher FR20 in resistant cells (two‐sided Wilcoxon Rank‐Sum test *p* < 2.2 × 10^−^^1^ ^6^, Figure 4E; Figure S4E). Bulk RNA‐seq or microarray data from six cell lines (Validation Cohorts II–VII) also supported higher FR20 in resistant cells (Figure 4F–H). In CCLE and GDSC (Validation Cohorts VIII–IX), high‐FR20 groups had higher AUC values (two‐sided Wilcoxon Rank‐Sum test *p* = 0.055 and 0.00076, Figure 4I), demonstrating FR20's predictive power for JQ1 response. These findings underscore FR20's utility in assessing JQ1 resistance.

**TABLE 1:** Summary of validation data cohorts used for FR20.

Dataset	Data source	Samples	Data Type	#Samples	Refs.
Validation Cohort I	GSE131135	SUM149 cell line (TNBC)	scRNA‐seq	1340	[22]
Validation Cohort II	GSE131135	SUM149 cell line (TNBC)	RNA‐seq	6	[22]
Validation Cohort III	GSE131135	SUM159 cell line (TNBC)	RNA‐seq	8	[22]
Validation Cohort IV	GSE164813	H23 cell line (lung adenocarcinoma)	microarray	6	[23]
Validation Cohort V	GSE164813	H1975 cell line (lung adenocarcinoma)	microarray	6	[23]
Validation Cohort VI	GSE103082	22RV1 cell line (prostate cancer)	RNA‐seq	3	NA
Validation Cohort VII	GSE103082	LNCaP cell line (prostate cancer)	RNA‐seq	3	NA
Validation Cohort VIII	CCLE	Cancer cell lines (pan‐cancer)	RNA‐seq	1019	[24]
Validation Cohort IX	GDSC	Cancer cell lines (pan‐cancer)	microarray	1018	[25]

### 2.4 The Heterogeneity of JQ1 Resistant Cells

**FIGURE 5:**
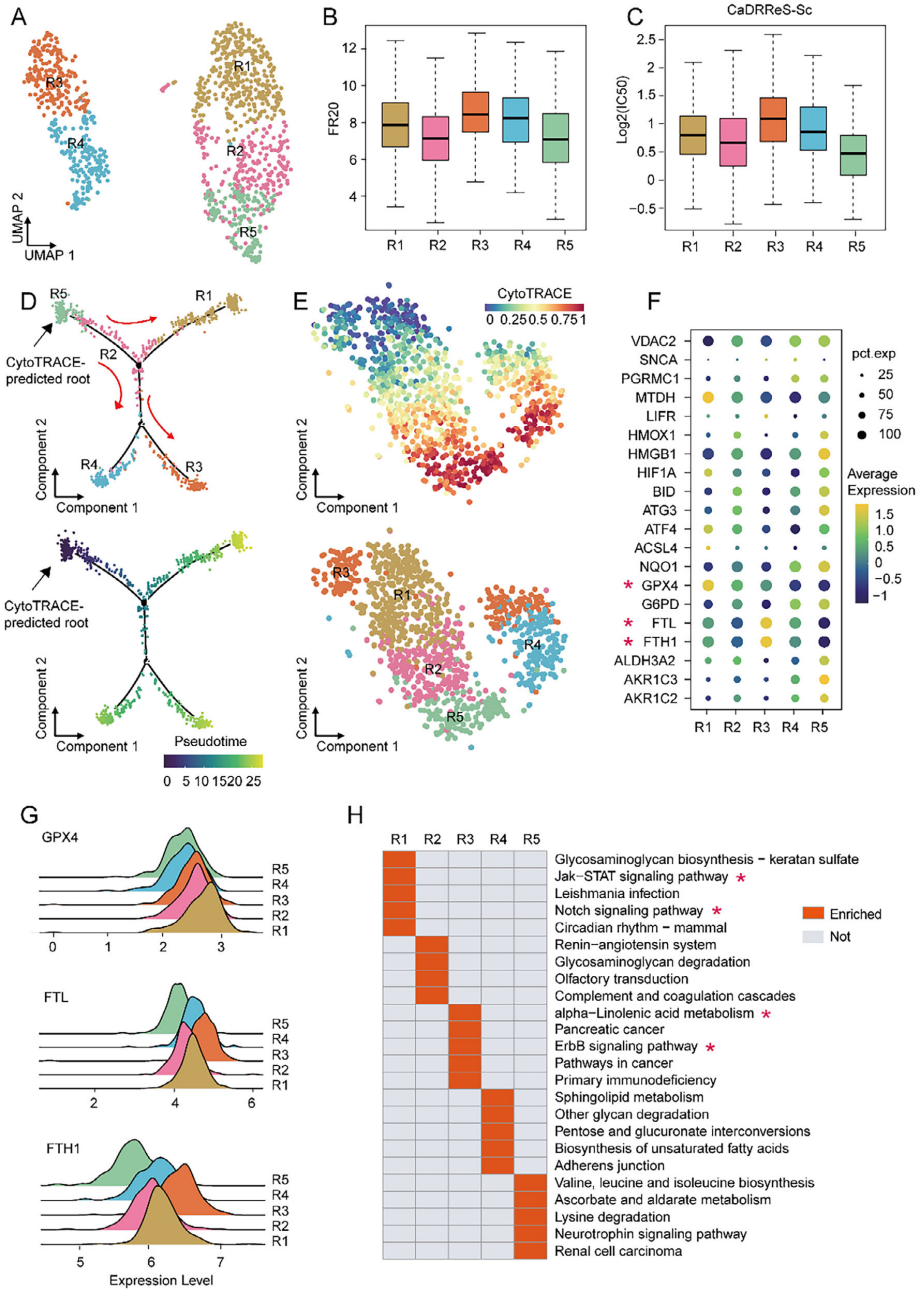
Heterogeneity of JQ1 resistant cells. (A) UMAP visualization of resistant cells at the resolution of 0.5. (B) Boxplots of FR20 score in five resistant sub‐clusters. (C) Boxplots of half‐maximal Inhibitory Concentration (IC50) for five resistant sub‐clusters predicted by CaDRReS‐Sc. (D) Lineage trajectory of resistant sub‐clusters inferred by Monocle2. Cells are labeled by sub‐cluster (top) or pseudotime (bottom). (E) CytoTRACE analysis of five sub‐clusters. Cells are labeled by CytoTRACE score (top) or sub‐cluster (bottom). (F) Dot heatmap of JQ1 resistance‐related signature in resistant sub‐clusters. (G) Ridgeplot of expression of *GPX*4, *FTL*, and *FTH*1 in resistant sub‐clusters. (H) Top five significantly and specifically up‐regulated KEGG pathways in each resistant sub‐cluster.

Resistant cells formed two distinct clusters (Figure 2A), indicating higher heterogeneity in resistant cells. Re‐clustering analysis for the resistant cells identified five sub‐clusters (R1, R2, R3, R4, and R5, Figure 5A) at the resolution of 0.5, determined by the largest silhouette score (Figure S5). JQ1 response varied significantly among sub‐clusters (ANOVA test *p* = 2.11 × 10^−18^, Figure 5B), with R3 showing the highest FR20 score and R5 the lowest. These results were further supported by CaDRReS‐Sc method [26] (Figure 5C). Pseudotime analysis revealed lineage relationships, with R5 as the “root” (Figure 5D,E; Figure S6), suggesting different stages of resistance evolution. Furthermore, the five sub‐clusters showed different expression patterns of the 20 ferroptosis regulators (Figure 5F). In general, R1 highly expressed *GPX*4 (log_2_ *FC* = 0.359, *p* = 2.074 × 10^−28^, Figure 5F,G); R3 highly expressed *FTH*1 (log_2_ *FC* = 0.53, *p* = 8.552 × 10^−46^) and *FTL* (log_2_ *FC* = 0.492, *p* = 1.121 × 10^−36^, Figure 5F,G); while R5 showed lower expression of *GPX*4 (log_2_ *FC* = −0.357, *p* = 2.509 × 10^−18^), *FTH*1 (log_2_ *FC* = −0.622, *p* = 1.584 × 10^−47^) and *FTL* (log_2_ *FC* = −0.561, *p* = 7.403×10^−40^, Figure 5F,G). Pathway analysis revealed functional differences: R1 was enriched in Jak‐STAT signaling pathway and Notch signaling pathway, while R3 was enriched in alpha‐Linolenic acid metabolism and ErbB signaling pathway (Figure 5H; Table S5). These findings indicated the heterogeneity in JQ1 resistant cells.

### 2.5 Drug Screening for JQ1 Re‐Sensitizer

**FIGURE 6:**
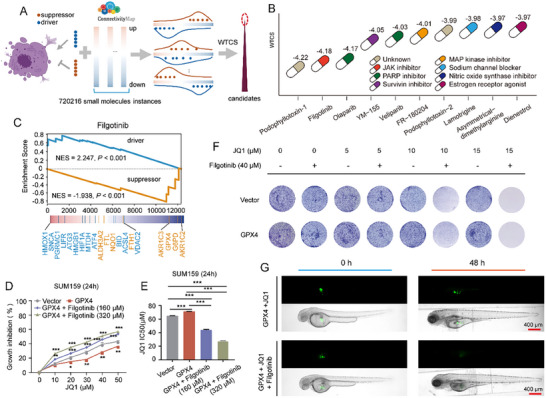
Prediction and validation of JQ1 re‐sensitizers. (A) Flowchart for identifying small molecules to reverse JQ1 resistance by D‐FR20 algorithm. (B) Top 10 candidate small molecules predicted by D‐FR20 for JQ1 re‐sensitizers. Color for each drug represents the mode of action (MOA). (C) Enrichment plot of 12 ferroptosis drivers and 8 ferroptosis suppressors in filgotinib treatment using GSEA. (D) CCK8 assay of JQ1 inhibition rate (24 h) in Vector and *GPX*4 overexpression groups (treated or untreated with fligotinib). (E) IC50 values of JQ1 (24 h) in Vector and *GPX*4 overexpression groups (treated or untreated with fligotinib). Significance in (D) and (E) was calculated using the one‐way ANOVA (n = 3). * *p* < 0.05; ** *p* < 0.01; *** *p* < 0.001. (F) Colony formation assays of SUM159 cells in Vector and *GPX*4 overexpression groups (treated or untreated with JQ1, fligotinib, or combined, respectively). (G) Representative fluorescence images of TNBC xenografts in zebrafish at 0 and 48 h post‐treatment, comparing *GPX*4‐overexpressing tumors between JQ1 monotherapy and JQ1 + filgotinib combination groups. Scale bar = 400 µm.

The FR20 score has demonstrated its ability to accurately reflect JQ1 resistance. Based on the CMap concept [15], we developed D‐FR20, an algorithm to identify drugs that restore JQ1 sensitivity by targeting the expression of 20 ferroptosis regulators. The algorithm prioritizes small molecules that inhibit up‐regulated ferroptosis suppressors and induce down‐regulated ferroptosis drivers. The WTCS [15, 27] was employed to merge *NES*_up_ and *NES*_down_ of each instance. A smaller WTCS indicates stronger potential for reversal (Figure 6A). As a result, the top 10 drugs were identified (Figure 6B; Table S6), including several anticancer agents. For example, Olaparib is an FDA‐approved (https://www.fda.gov/) PARP inhibitor for cancer treatment including breast, ovarian, pancreatic, and prostate cancers. Podophyllotoxin derivative has been shown to increase apoptosis and decreases tumor burden in TNBC cells by directly activating AMPK and modulating the Warburg effect, suggesting the anticancer potential of podophyllotoxin [28]. Veliparib, another PARP inhibitor, has shown potential in TNBC treatment by combinations with Carboplatin [29].

To validate D‐FR20, we conducted both in vitro and in vivo experiments. Among the top 10 candidates (Figure 6B; Table S6), filgotinib (a selective JAK1 inhibitor ranked second) effectively reversed the dysregulation of 16 ferroptosis‐related regulators in JQ1‐resistant cells, including *GPX*4 (Figure 6C; Figure S7A). Cell viability assays demonstrated that filgotinib reduced *GPX*4 levels and restored JQ1 sensitivity in TNBC cells (Figure 6D,E; Figure S7B–I). This effect was further corroborated by colony formation assays, which confirmed filgotinib's ability to re‐sensitize TNBC cells to JQ1 (Figure 6F; Figure S8). To extend these findings in vivo, we employed a zebrafish xenograft model. Consistent with the in vitro results, filgotinib treatment significantly restored JQ1 sensitivity in TNBC cells (Figure 6G; Figures S9 and S10). Collectively, these results validate the predictive power of D‐FR20 and highlight filgotinib as a promising therapeutic candidate for overcoming JQ1 resistance in TNBC.

## 3 Discussion

The emergence of resistance to BBDIs represents a major barrier to their clinical application in TNBC. While scRNA‐seq has provided unprecedented resolution to study tumor heterogeneity [6, 7], a systematic framework that connects dynamic transcriptional reprogramming, specific resistance mechanisms, and actionable therapeutic strategies is still lacking. In this study, we developed and validated an integrated computational‐experimental pipeline that not only delineates the molecular landscape of JQ1 resistance but also provides practical tools for its quantification and therapeutic targeting.

Our longitudinal scRNA‐seq analysis first revealed a coordinated reprogramming of TNBC cells during the acquisition of JQ1 resistance. We observed a clear increase in transcriptional heterogeneity, with resistant cells segregating into distinct clusters that were absent in the sensitive population (Figure 2A). Analysis of dynamic changes in gene expression programs revealed a consistently up‐regulated pattern (Pattern 3) that showed significant enrichment for ferroptosis‐related pathways (Figure 2C,D). Concurrent phenotypic shifts included a reduction in the basal‐like subtype proportion (Figure 2E) and an increase in G1‐phase arrested cells (Figure 2F), consistent with JQ1's known effects on cell cycle progression [18, 19]. Pseudotime analysis further reconstructed the evolutionary trajectory from sensitive to resistant states (Figure 2G), providing a temporal framework for resistance development.

Crucially, our computational analyses implicated ferroptosis suppression as a potential resistance mechanism. Differential expression analysis revealed a significant upregulation of the ferroptosis suppressor *GPX*4 in resistant cells (Figure 3A), and pathway enrichment analysis further identified ferroptosis as one of the most prominently dysregulated pathways (Figure 3B). To move beyond correlation and establish causation, we performed functional validation. Overexpression of *GPX*4 induced JQ1 resistance, reflected by decreased inhibition rates and increased IC50 values (Figure 3D,E), whereas *GPX*4 knockdown effectively restored drug sensitivity in resistant cells (Figure 3F,G). These findings were further corroborated by in vitro colony formation assays (Figure 6F; Figure S8) and in vivo models (Figures S9 and S10). Collectively, these results establish *GPX*4 as a functional contributor to the resistant phenotype and suggest ferroptosis suppression as a potential mechanism involved in BET inhibitor resistance.

To translate these mechanistic insights into an applicable metric, we developed the FR20 score, which integrates the expression of 20 dysregulated ferroptosis regulators. FR20 successfully distinguished resistant from sensitive cells in our discovery cohort (Figure 4D) and demonstrated remarkable robustness across nine independent validation datasets encompassing both single‐cell and bulk transcriptomic profiles (Figure 4E–I, Table 1). Beyond binary classification, FR20 revealed previously unappreciated heterogeneity within the resistant population. Resistant cells segregated into five sub‐clusters with significantly different FR20 scores (ANOVA *p* = 2.11 × 10^−^^1^ ^8^, Figure 5B). This stratification pattern and the associated gradient of drug resistance were consistently validated by another algorithm CaDRReS‐Sc [26] (Figure 5C). This heterogeneity was underpinned by distinct expression patterns of ferroptosis regulators (Figure 5F,G) and enriched pathways (Figure 5H), suggesting diverse adaptive survival strategies among resistant cells — a finding crucial for understanding treatment failure [9, 10]. Building upon the FR20 framework, we then developed D‐FR20 to systematically screen for compounds capable of reversing the ferroptosis‐related resistance signature. This approach identified filgotinib, a selective JAK1 inhibitor, as a top candidate re‐sensitizer (Figure 6A,B). Validation formed a multi‐layered evidentiary chain: (1) Mechanistically, filgotinib treatment reversed the expression of key ferroptosis regulators, including *GPX*4, in resistant cells (Figure 6C); (2) In vitro, it effectively restored JQ1 sensitivity in both cell viability assays (Figure 6D,E) and colony formation assays (Figure 6F); (3) In vivo, the filgotinib‐JQ1 combination significantly inhibited tumor growth in a zebrafish xenograft model of *GPX*4‐overexpressing TNBC (Figure 6G). These results collectively validate D‐FR20's predictive power and establish filgotinib as a promising combination therapy to overcome BET inhibitor resistance.

Our findings on the transcriptional reprogramming of ferroptosis genes can be further contextualized within the known epigenetic mechanism of JQ1. As a BET inhibitor, JQ1 functions by displacing BRD4 from chromatin, thereby altering the epigenetic landscape and downstream gene expression [4]. In line with this, we observed that JQ1 treatment generally inhibited the binding affinity of BRD4 to ferroptosis‐related genes in sensitive cells (Figure S11). Intriguingly, this inhibitory effect was significantly attenuated in resistant cells (Figure S11). These results suggest that acquired resistance may involve a compensatory adaptation at the epigenetic level, potentially mitigating JQ1's core action of disrupting BET protein occupancy at key survival gene loci, such as those regulating ferroptosis. This epigenetic plasticity could contribute to the stable transcriptional reprogramming captured by our scRNA‐seq analysis.

While our study provides compelling evidence for ferroptosis‐mediated resistance and identifies a potential therapeutic strategy, several limitations warrant consideration. First, our analyses primarily utilized cell line models; future incorporation of patient‐derived samples would strengthen clinical translation [11]. Second, although we identified transcriptional dysregulation of ferroptosis genes and validated the functional role of *GPX*4, the upstream regulatory mechanisms — such as potential epigenetic modifications or signaling pathway alterations — require further in‐depth exploration and validation. Third, the efficacy and safety of filgotinib‐JQ1 combination therapy require further validation in more complex preclinical models. Nevertheless, our integrated computational‐experimental framework offers a generalizable approach for studying drug resistance and identifying combination therapies across cancer types.

In summary, this study establishes a cohesive narrative from molecular discovery to therapeutic application. We leveraged scRNA‐seq to identify ferroptosis inhibition as a potential mechanism of BET inhibitor resistance, encapsulated this mechanism into a quantitative biomarker (FR20) for resistance prediction and heterogeneity analysis, and employed a signature‐based algorithm (D‐FR20) to identify and validate a novel re‐sensitizing agent. Our work provides not only mechanistic insights into BET inhibitor resistance but also a pair of computational tools and a candidate therapeutic strategy, offering a tangible path forward for addressing this clinical challenge in TNBC treatment.

## 4 Materials and Methods

### 4.1 TNBC scRNA‐Seq Data Collection and Analysis

The scRNA‐seq UMI count of SUM159 TNBC cell lines in four stages, including parental (DMSO, sensitive), JQ1‐treated (JQ1) and JQ1 resistant (RDMSO), as well as JQ1 resistant cells retreated with JQ1 (RJQ1), were downloaded from GEO (https://www.ncbi.nlm.nih.gov/geo/) database (GSE131135) [22]. The Seurat R package (V 4.3.0) was utilized for quality control and downstream analysis [30]. Genes expressed in fewer than three cells were removed, and cells covering fewer than 200 genes or more than 20% UMIs from mitochondrial genes were filtered out. Data was normalized using the NormalizeData function, and the top 2000 highly variable genes were selected using the FindVariableGenes function. Principal component analysis (PCA) was performed on the top 10 dimensions, followed by visualization using uniform manifold approximation and projection (UMAP). Unsupervised clustering analysis was conducted using the FindClusters function. To explore resistant cell heterogeneity, re‐clustering analysis was performed, and the optimal resolution was determined using cluster silhouette scores. The differentially expressed genes (DEGs) were identified using the FindMarkers function with criteria of log‐transformed fold chang (log_2_ *FC*) > 0.25 and *FDR* < 0.01.

### 4.2 Identification of Dynamic Expression Programs

In order to explore the dynamic expression programs across the four stages, all DEGs at each stage were clustered by the fuzzy c‐means clustering method, which was performed by Mfuzz R package [31]. First, the average of gene expression in each stage was calculated, followed by “filter.std (min.std = 0)”, “standardize()”, and “mestimate()” functions with default parameters. Then, the DEGs were clustered into six different expression programs with cluster membership > 0.6.

### 4.3 Gene Set Enrichment Analysis

To investigate the biological pathways related to JQ1 resistance, KEGG pathway enrichment analysis was performed using the enrichKEGG function of the clusterProfiler R package (V 4.8.1) [32] and Metascape [33]. To compare the enriched KEGG pathways of the resistant sub‐clusters, Gene set variation analysis (GSVA) was performed to calculate the gene set enrichment scores using “ssGSEA” method [34]. The difference in gene set enrichment scores between each sub‐cluster and all other sub‐clusters was evaluated by the Wilcoxon Rank‐Sum test. The top five significantly up‐regulated pathways in each sub‐cluster were shown (*p* < 0.05).

### 4.4 PAM50 Subtyping and Cell Cycle Assignment

The PAM50 subtyping algorithm, based on the expression of 50 signature genes, was used to classify breast cancer cells into five molecular subtypes (Luminal A, Luminal B, HER2‐enriched, Basal‐like, and Normal‐like). The PAM50 subtype of each single cell was calculated using the genefu R package [17]. Cell cycle phases were assigned using the Seurat CellCycleScoring function [30].

### 4.5 Cell Lineage Trajectory Inference

Monocle2 (V 2.18.0) [35] was employed to infer the lineage relationships among the four stages and the resistant sub‐clusters of TNBC cells. CytoTRACE [36] was used to predict the “root” (starting point) of the evolution process. To obtain the starting point of resistant sub‐clusters, CytoTRACE and Monocle2 algorithms with the default parameters were used to construct resistant sub‐cluster pseudo‐trajectories.

### 4.6 Single Cell Copy Number Variation (CNV) Analysis

The CNVs for each cell were estimated by inferCNV R package (V 1.6.0) [37]. The normal human 293T cells were downloaded from 10x website (https://support.10xgenomics.com/single‐cell‐gene‐expression/datasets/1.1.0/293t) and used to derive the baseline reference.

### 4.7 Gene Regulatory Network Construction

PySCENIC was used for constructing gene regulatory networks and evaluate transcription factor (TF) activity [21]. The two‐sided Wilcoxon Rank‐Sum test was utilized to identify significantly differentially activated TFs (*p* < 0.01) between resistant and sensitive cells.

### 4.8 FR20 Score for Estimating Resistant Risk

We obtained the ferroptosis regulators, including 264 drivers and 238 suppressors, from FerrDb V2 [38]. The ferroptosis drivers that were significantly down‐regulated in resistant cells and the ferroptosis suppressor that were significantly up‐regulated in resistant cells were considered as JQ1 resistance‐related signature (n = 20). The resistant risk score (FR20) was calculated as:

$$FR20=\sum_{i=1}^{n}w_{i}\times Exp_{i}$$

where *w*_i_ =  1 for suppressors, *w*_i_ =   − 1 for drivers. *Exp*_i_ is the normalized expression of gene *i*.

### 4.9 Validation of FR20 in Multiple Cohorts

For SUM159 scRNA‐seq data (Discovery Cohort), the FR20 score was compared between the induced JQ1‐resistant cells and sensitive cells. Then, we used nine independent datasets, named Validation Cohort I‐IX, to validate the accuracy and scalability of FR20. For the JQ1 resistant and sensitive cells, we first calculated the FR20 scores for each cell, and then made a comparison between resistant and sensitive cells. In addition, we also validated FR20 for bulk RNA‐seq or microarray data. FR20 score was calculated for all of the six induced JQ1‐resistant cell lines and the sensitive cell lines. Two‐tailed t‐test was used to compare FR20 in cell lines SUM159 (Validation Cohort II), SUM149 (Validation Cohort III), H23 (Validation Cohort IV), H1975 (Validation Cohort V). Mean FR20 score was used in comparison because of lacking enough replicates in 22RV1 (Validation Cohort VI) and LNCaP (Validation Cohort VII). For the cell lines with drug response data (area under of dose response curve, AUC) in Cancer Cell Line Encyclopedia (CCLE) (Validation Cohort VIII) and Genomics of Drug Sensitivity in Cancer (GDSC) (Validation Cohort IX), we defined the cell lines in top and bottom quartile FR20 scores as high‐FR20 and low‐FR20 groups, respectively. Then, we compared the difference of AUC values between high‐FR20 and low‐FR20 groups (two‐sided Wilcoxon Rank‐Sum test).

### 4.10 D‐FR20 Algorithm for Screening Potential Drugs Reversing JQ1 Resistance

To identify potential drugs capable of reversing JQ1 resistance, we proposed an algorithm, named D‐FR20, to investigate whether the up‐regulated ferroptosis suppressors (or down‐regulated ferroptosis drivers) was enriched in the bottom (or top) of the rank‐ordered signature of drug perturbations. First, we obtained drug‐perturbed gene expression profiles of 720216 small molecule instances from LINCS. For each instance, we calculated the normalized enrichment score (NES) for both the up‐regulated ferroptosis suppressor genes and the down‐regulated ferroptosis driver genes by Gene Set Enrichment Analysis (GSEA) [39] using gseapy [40] with 1000 permutations. We retained the significantly enriched small molecules (*FDR* < 0.05) for both up‐regulated ferroptosis suppressors and down‐regulated ferroptosis drivers. Finally, we employed the weighted connectivity score (WTCS) to reflect the re‐sensitizing level of candidate small molecules [15, 27].

$$\mathrm{WTCS}\;=\left\{\;\begin{array}{c} \left(\mathrm{NE}S_{\mathrm{up}}-\mathrm{NE}S_{\mathrm{down}}\right)/2,\;\mathrm{ifsi}\mathrm{gn}\left(\mathrm{NE}S_{\mathrm{up}}\right)\neq \mathrm{sign}\left(\mathrm{NE}S_{\mathrm{down}}\right)\\ 0,\;\mathrm{othe}\mathrm{rwise} \end{array}\right.$$

where *NES*_up_ is for suppressors and *NES*_down_ for drivers. The top 10 small molecules ranked by WTCS from low to high were regarded as candidates.

### 4.11 Statistical Analysis

All statistical analyses were performed using R (version 4.3.0) and GraphPad Prism (version 9.0). Data are presented as mean ± standard deviation (SD) unless otherwise specified. Statistical significance was defined as *p* < 0.05 unless otherwise specified. All sample sizes (n) and specific statistical tests used for each experiment are also detailed in the corresponding figure legends and results sections. The specific statistical tests used in this study are as follows:

1) DEGs between groups were identified using the FindMarkers function in the Seurat R package (version 4.3.0) with criteria of log_2_ *FC* > 0.25 and *FDR* < 0.01.
2) KEGG pathway enrichment was performed using the enrichKEGG function of the clusterProfiler R package (version 4.8.1) and Metascape. GSVA was performed using the “ssGSEA” method to calculate enrichment scores. *P* < 0.05 was considered statistically significant.
3) Significant differences in copy number variation (CNV) levels and transcription factor (TF) activities between resistant and sensitive cells were assessed using the two‐sided Wilcoxon Rank‐Sum test. *P* < 0.05 for CNV and *p* < 0.01 for TF activity were considered statistically significant, respectively.
4) The validation of FR20 scores across diverse datasets employed tailored statistical approaches. For single‐cell resolution data (Discovery Cohort and Validation Cohort I), differences in FR20 scores between resistant and sensitive cell populations were assessed using two‐sided Wilcoxon Rank‐Sum test. In bulk transcriptomic datasets (Validation Cohorts II–VII), a two‐tailed t‐test was applied to compare FR20 scores between resistant and sensitive cell lines (SUM159, SUM149, H23, H1975). For the 22RV1 and LNCaP datasets, which contained limited biological replicates, group‐wise comparisons were based on mean FR20 scores. For pan‐cancer cell lines (Validation Cohorts VIII–IX), a two‐sided Wilcoxon Rank‐Sum test was used to evaluate differences in drug‐response AUC values between cell lines stratified into high‐ and low‐FR20 groups. *P* < 0.05 was considered statistically significant.
5) To characterize heterogeneity within JQ1‐resistant cell populations, we employed multiple statistical approaches. Differences in FR20 scores among the five identified resistant sub‐clusters were assessed using ANOVA with a significance threshold of *p* < 0.05. Subsequently, differences in gene set enrichment scores between each resistant sub‐cluster and all other sub‐clusters were evaluated using the Wilcoxon Rank‐Sum test, with a significance threshold of *p* < 0.05.
6) For *GPX*4 overexpression experiments assessing JQ1 inhibition rates and IC50 values, significance was determined using unpaired two‐tailed Student's t‐tests (n = 3). For *GPX*4 knockdown experiments, one‐way ANOVA was applied (n = 3). *P* < 0.05 was considered statistically significant.
7) The D‐FR20 algorithm for screening potential JQ1 re‐sensitizers was implemented as follows. NES for drug perturbations were calculated using GSEA executed via gseapy with 1000 permutations. *FDR* < 0.05 was considered statistically significant.
8) In the filgotinib re‐sensitization experiments assessing JQ1 inhibition rates and IC50 values, one‐way ANOVA was applied (n = 3). *P* < 0.05 was considered statistically significant.

## Author Contributions

W.J. and L.H.W. designed the study; H.Z.L. and M.Q.Y carried out data acquisition and analysis and drafted the manuscript; Y.N.S. performed the low‐throughput experiment validation. J.H.C. and F.H. performed data curation and investigation. All authors read and approved the final manuscript.

## Funding

This work was supported by the National Natural Science Foundation of China (62472095, 62172213, and 81972478), the Natural Science Foundation of Fujian Province of China (2024J08164), the Fujian Provincial Health and Wellness Science and Technology Plan Project (2024GGA037 and 2024QNA036), the Joint Funds for the Innovation of Science and Technology, Fujian Province (2024Y9151 and 2024Y9121).

## Conflicts of Interest

The authors declare no conflicts of interest.

## Supporting information




**Supporting File 1**: advs74761‐sup‐0001‐SuppMat.pdf.

**Supporting File 2**: advs74761‐sup‐0002‐TableS1.xlsx.

**Supporting File 3**: advs74761‐sup‐0003‐TableS2.xlsx.

**Supporting File 4**: advs74761‐sup‐0004‐TableS3.xlsx.

**Supporting File 5**: advs74761‐sup‐0005‐TableS4.xlsx.

**Supporting File 6**: advs74761‐sup‐0006‐TableS5.xlsx.

**Supporting File 7**: advs74761‐sup‐0007‐TableS6.xlsx.

## Data Availability

All data used in this study could be obtained from public sources and were described at the relevant locations within the text. All codes used in this study are available at https://github.com/mengqyuan/BBDI_resistance.
