# Supplementary material for: Single‐Cell Computational Frameworks for Quantifying BET Bromodomain Inhibitor Resistance and Screening Re‐Sensitizer Drugs in Triple‐Negative Breast Cancer
Source: Adv Sci (Weinh). 2026 Apr 3;13(29):e13246. doi: 10.1002/advs.202513246 (PMC13205605; doi:10.1002/advs.202513246)
Supplement: Supplementary file 1 — Supporting File 1: advs74761‐sup‐0001‐SuppMat.pdf. [file ADVS-13-e13246-s004.pdf]

Supplementary Materials

Supplementary Methods

Cell culture

The human triple-negative breast cancer cell line SUM159 (RRID: CVCL_5423) was obtained from the Shanghai Institutes for Biological Sciences, Chinese Academy of Sciences. Cells were maintained in Ham's F-12 medium (Gibco, USA) supplemented with 5% fetal bovine serum (FBS), 5 μg/mL insulin, and 1 μg/mL hydrocortisone. Cultures were incubated at 37°C in a humidified atmosphere containing 5% CO_2. Regular testing confirmed the cell line was free from mycoplasma and other contaminants.

CCK-8 Assay

SUM159 cells were seeded into 96-well plates and allowed to adhere for 24 hours. Following this, the original medium in each well was replaced with a medium containing the designated drug mixture. After either 24 or 48 hours, wells were treated with CCK-8 solution and the plates were incubated at 37°C for 3.5 hours. Optical density (OD) values were measured using an enzyme-linked immunosorbent assay (ELISA) reader and subsequently analyzed systematically to determine the rate of cell inhibition. All experiments were performed in triplicate.

Transfection Procedure

SUM159 cells were plated in 6-well plates and incubated overnight. Subsequently, the medium was replaced with fresh culture medium, supplemented with specified quantities of the GV513 lentiviral vector with GFP fluorescence to overexpress *GPX*4 (GenePharma, China). For *GPX*4 knockdown, the SUM159 cells were transfected with siRNA and riboFECTTM CP Reagent (RiboBio, China) in 24-well plates for 24 hours. The efficacy of the transfection was evaluated using RT-qPCR and Western blotting.

Colony formation assay

SUM159 cells were seeded at a density of 800 cells per well in 6-well plates and cultured for 10 days. Then, the cells were fixed with 1 ml of methanol per well and stained with a 0.1% crystal violet solution. Subsequently, photographs of the stained colonies were captured for documentation and analysis. Image J software is used to perform quantitative analysis of colony results.

RT-qPCR analysis

Total RNA was isolated by FastPure Cell/Tissue Total RNA Isolation Kit V2 reagent (Vazyme Biotech Co., Ltd). One microgram of RNA was used to synthesize cDNA using the HiScripteIII RT SuperMix for qPCR (Vazyme Biotech Co., Ltd). Real-time PCR was performed using SYBR Green real-time PCR analysis (Vazyme Biotech Co., Ltd) with the specific primers. The primers for *GPX*4 are F： 5'-CCCAGTGAGGCAAGACCGAAG-3' and R: 5'-GGCTCCTGCTTCCCGAACTG-3', respectively. And the primers of housekeeping gene GAPDH were F: 5'-CGGAGTCAACGGATTTGGTCG-3' and R: 5'-TCTCGCTCCTGGAAGATGGTGAT-3', respectively. PCR results, recorded as cycle threshold (Ct), were normalized against the internal control (GAPDH).

Western blot

The Western blot procedure began with cell collection and lysis. The lysates were subsequently centrifuged at 4°C at a speed of 10,000 rpm for 10 minutes. The resulting supernatant, containing the protein lysate, was stored at -80°C until further use. Total protein concentration in these lysates was quantified using the BCA Protein Assay Kit (Beyotime, China). Equal amounts of protein from each sample were subjected to sodium dodecyl sulfate-polyacrylamide gel electrophoresis (SDS-PAGE) using gels provided by Beyotime (China). Following electrophoresis, proteins were transferred onto polyvinylidene fluoride (PVDF) membranes (Millipore, USA). To

block non-specific binding, the membranes were incubated with 5% skimmed milk. Subsequently, they were incubated overnight at 4°C in a diluent containing primary antibodies. The primary antibodies used were GPX4 at a dilution of 1:5000 (Abcam, USA) and GAPDH at a dilution of 1:20000 (Proteintech, USA), serving as a loading control. Subsequently, the membranes were incubated with a secondary antibody at a dilution of 1:10000 (Biosharp, China) for 1 hour at room temperature. The protein bands of interest were visualized using a chemiluminescent substrate and photographed with a luminescent image analyzer. The resulting images were then analyzed using Image J software for the quantification and comparison of protein expression levels across different samples.

Zebrafish culture and experiments

The zebrafish used in this study were AB strain zebrafish, propagated by Guangdong Longseek Testing Co., Ltd. Six experimental groups were established as follows: SUM159 group, SUM159 + JQ1 (1 μM) group, *GPX*4 overexpression group, *GPX*4 overexpression + JQ1 (1 μM) group, *GPX*4 overexpression + Figotinib group (25 μM), *GPX*4 overexpression + JQ1 (1 μM) + Figotinib (25 μM) group. Healthy zebrafish embryos at 48 hours post-fertilization (hpf) were selected and randomly distributed onto agar plates. Control SUM159 with GFP fluorescence or *GPX*4-overexpressed tumor cells with GFP fluorescence were microinjected into the yolk sac of each embryo using a micromanipulator-equipped microinjection system. Following injection, the embryos were incubated in a temperature-controlled incubator maintained at 28.5 °C for 1 hour. After incubation, zebrafish embryos exhibiting tumor cells of comparable size and no signs of metastasis were selected under a fluorescence microscope for subsequent experiments.

The selected zebrafish were transferred to cell culture plates. The SUM159 and *GPX*4 overexpression groups were supplemented with E3 embryo medium, while the remaining groups received their respective drug treatments. All groups were incubated at 28.5 °C in a temperature-controlled incubator. Tumor cell proliferation was

assessed by observing and photographing the embryos under a fluorescence microscope at 0 hours and 48 hours post-intervention. Fluorescence intensity (*FI*) value occupied by tumor cells within the zebrafish was quantified using ImageJ software. For each zebrafish, the magnitude of change of fluorescence intensity (CFI) in tumor cell from 0 to 48 hours was calculated as follows:

$$CFI = \frac{FI(0h) - FI(48h)}{FI(0h)}$$

The zebrafish experiment protocol has been reviewed by the Animal Welfare and Ethics Committee of Guangdong LongSeek Testing Co., LTD, and it complies with the principles of animal protection, animal welfare, and ethics, as well as the relevant regulations of national animal welfare and ethics (IACUC LS 1202-01-2024).

Supplementary Results

***In vitro* experiments confirmed that filgotinib re-sensitizes TNBC cells to JQ1.**

To confim that filgotinib could re-sensitize TNBC cells to JQ1, we performed *in vitro* experiments. Initially, we examined the influence of filgotinib on the mRNA and protein levels of *GPX*4. Filgotinib treatment for 24 hours significantly reduced both the mRNA and protein levels of *GPX*4 in Vector and *GPX*4 overexpression groups (Figs. S7B-E). Nevertheless, the cell viability in both groups exhibited negligible impact under high-dose filgotinib treatment for 24 and 48 hours (Figs. S7F and S7G), indicating minimal cytotoxic effects on TNBC cells. Intriguingly, when used in combination with JQ1, cells overexpression *GPX*4 exhibited restored sensitivity to JQ1. The combination of filgotinib (160 μM and 320 μM) with JQ1 for 24 and 48 hours demonstrated a markedly improved growth inhibition, accompanied by a reduction in JQ1 IC50 value in *GPX*4 overexpression groups (Figs. 6D, 6E, S7H and S7I). These results showed the potential of filgotinib as JQ1 re-sensitizer in TNBC.

To further support these results, colony formation assays were conducted (Fig. 6F). The *GPX*4 overexpression groups had higher number of cell colonies under JQ1 treatment (5 μM, 10 μM and 15 μM) (Fig. S8A), also indicating the overexpression of

*GPX*4 induced JQ1 resistance. Filgotinib (40 μM) treatment alone did not significantly alter the colony number (Fig. S8B), however, the combination of JQ1 (5 μM, 10 μM and 15 μM) and filgotinib (40 μM) treatment resulted in a notably reduced colony number compared to JQ1 alone in *GPX*4 overexpression groups (Figs. S8C-E). In summary, our study identified the small molecule filgotinib as a potential enhancer of JQ1 sensitivity in resistant cells, offering novel insights for developing alternative therapeutic approaches to circumvent JQ1 resistance in TNBC.

***In vivo* experiments confirmed that filgotinib re-sensitizes TNBC cells to JQ1.**
We further validated the re-sensitizing effect of filgotinib using a zebrafish xenograft model with transplanted SUM159 cells or *GPX*4-overexpressed SUM159 cells (Figs. 6G and S9). After 48 hours of treatment with JQ1, filgotinib, or their combination, we measured the fluorescence intensity of tumors (Fig. S9). JQ1 treatment significantly reduced fluorescence intensity of SUM159 tumors, whereas *GPX*4 overexpression did not produce a significant change (Fig. S10A), suggesting that *GPX*4 overexpression confers resistance to JQ1. In the *GPX*4 overexpression group, filgotinib alone did not significantly decrease fluorescence intensity of tumors after 48 hours (Fig. S10A). However, the combination of filgotinib and JQ1 significantly reduced fluorescence intensity (Fig. S10A), indicating filgotinib's ability to re-sensitize cells to JQ1.

Additionally, we compared the magnitude of change of fluorescence intensity (CFI) in tumors from 0 to 48 hours across different groups (Fig. S10B). The JQ1 group exhibited significantly greater inhibition than the SUM159 group after 48 hours, demonstrating JQ1's efficacy under normal conditions. Conversely, the *GPX*4 overexpression + JQ1 group did not show significantly greater inhibition than the *GPX*4 overexpression group and had significantly lower inhibition than the SUM159 + JQ1 group, further confirming that *GPX*4 overexpression induces resistance to JQ1. *GPX*4 + filgotinib did not not show significantly greater inhibition than *GPX*4 group, suggesting its limited efficacy as a single agent. In contrast, the combination of filgotinib and JQ1 significantly enhanced inhibition compared to both the *GPX*4

overexpression group and the *GPX4* overexpression + JQ1 group, demonstrating filgotinib's role in overcoming JQ1 resistance.

In conclusion, our study identifies filgotinib as a potential enhancer of JQ1 sensitivity in resistant TNBC cells. Both *in vitro* and *in vivo* experiments demonstrate that filgotinib effectively re-sensitizes TNBC cells to JQ1. These findings offer novel insights for developing alternative therapeutic approaches to circumvent JQ1 resistance in TNBC.

Supplementary Figures

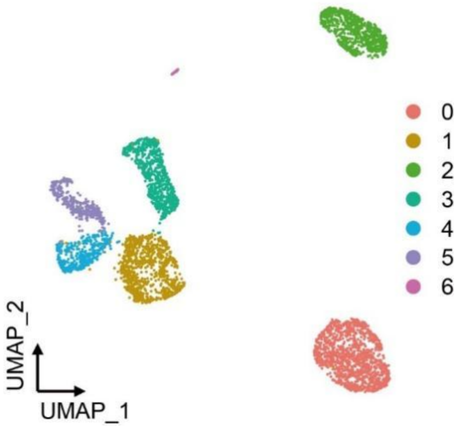

Figure S1. Uniform manifold approximation and projection (UMAP) visualization of SUM159 cells colored by clusters at the resolution of 0.3.

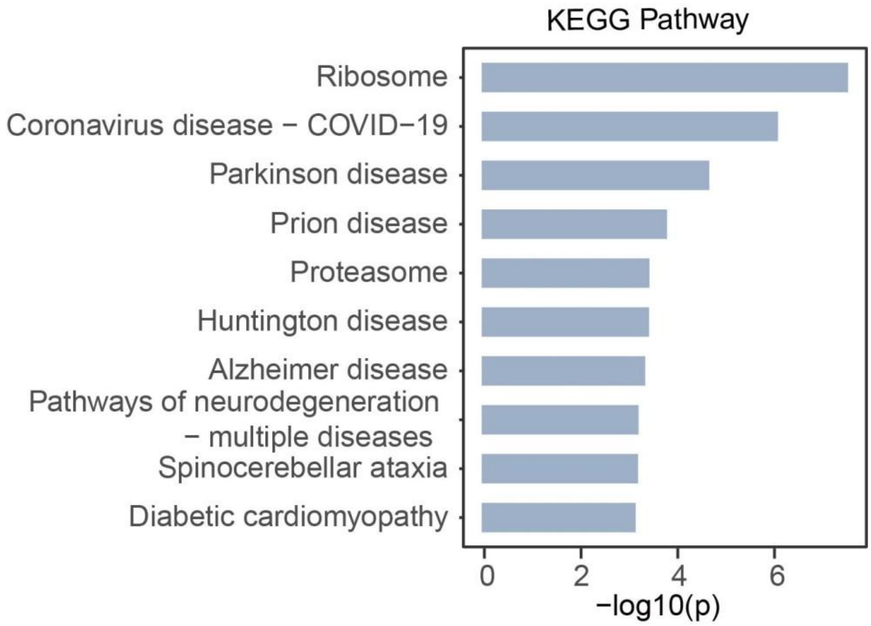

Figure S2. Bar graph of the top 10 KEGG pathways enriched in genes of pattern 4.

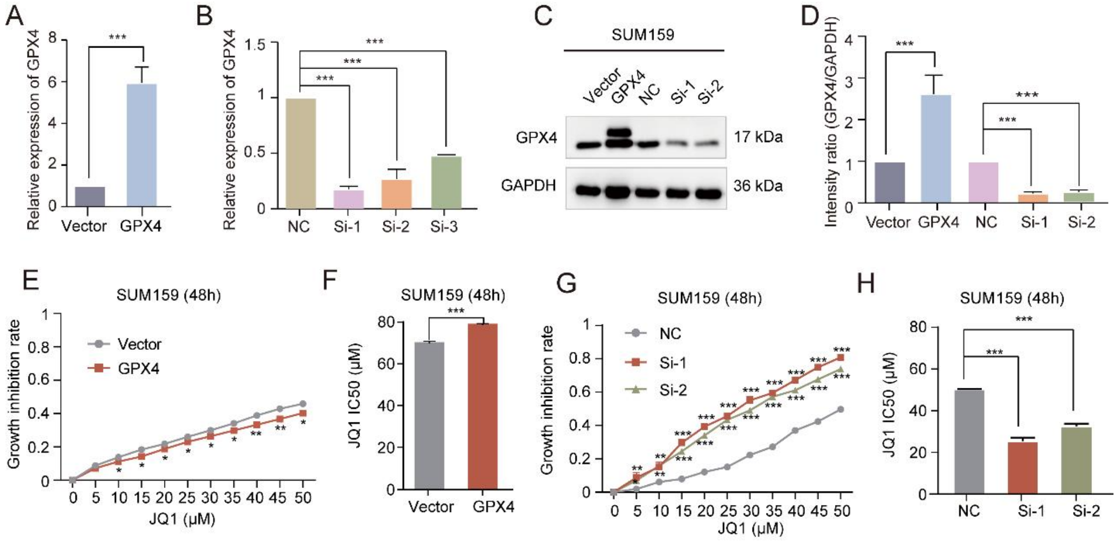

Figure S3. JQ1 efficacy is modulated by *GPX*4 expression levels. **A**. Relative mRNA expression of *GPX*4 in *GPX*4 overexpression group versus Vector control group. **B**. Relative mRNA expression of *GPX*4 in *GPX*4 knockdown group versus Vector control group. **C**. Western Blotting results of *GPX*4 overexpression and knockdown groups. **D**. Western Blotting quantitative results of *GPX*4 overexpression and knockdown groups. **E**. CCK8 assay results for JQ1 inhibition rate (48 h) in *GPX*4 overexpression group versus Vector control group. **F**. IC50 values of JQ1 (48 h) in *GPX*4 overexpression groups versus Vector control group. **G**. CCK8 assay results for JQ1 inhibition rate (48 h) in *GPX*4 knockdown groups versus Vector control group. **H**. IC50 values of JQ1 (48 h) in *GPX*4 knockdown groups versus Vector control group. Data represent mean ± SD, Significance in (**A**) and (**E**-**F**) was calculated using the Unpaired-samples T tests (n=3). Significance in (**B**, **D**) and (**G**-**H**) was calculated using the one-way ANOVA (n=3). * $p < 0.05$; ** $p < 0.01$; *** $p < 0.001$.

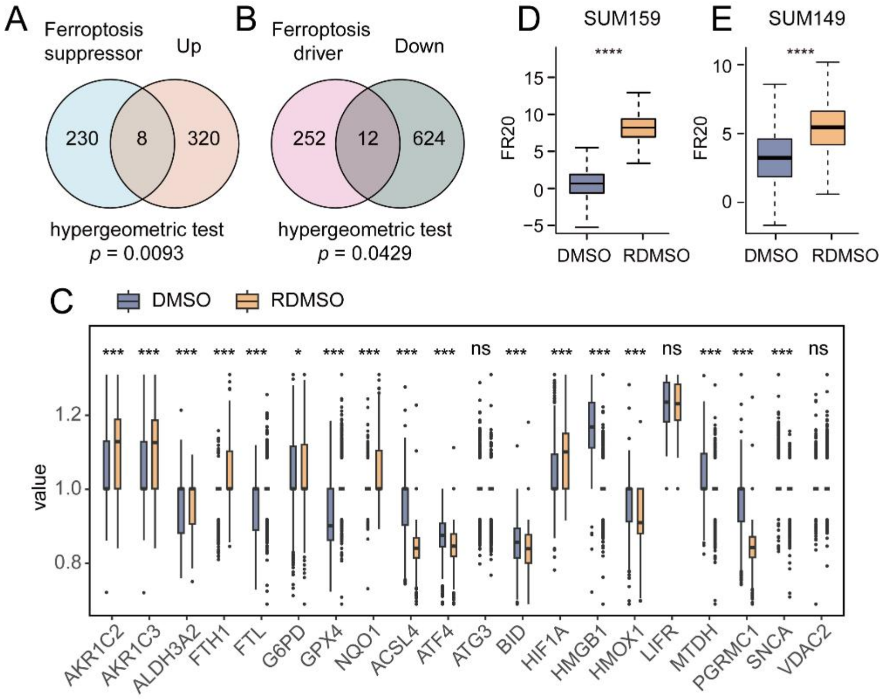

Figure S4. Dysregulation of 20 ferroptosis regulators in JQ1 resistance. **A**. Venn diagrams showed the overlapped genes between ferroptosis suppressors and significantly up-regulated genes in resistant cells. **B**. Venn diagrams showed the overlapped genes between ferroptosis drivers and significantly down-regulated genes in resistant cells. **C**. Boxplot of CNV values inferred by inferCNV. **D**-**E**. Boxplots of FR20 for single cells in JQ1 resistant and sensitive cells of SUM159 and SUM149 cell lines. The significance of the differences was determined using the two-sided Wilcoxon Rank-Sum test. ****: $p < 0.0001$, ***: $p < 0.001$; **: $p < 0.01$; *: $p < 0.05$; ns: not significant.

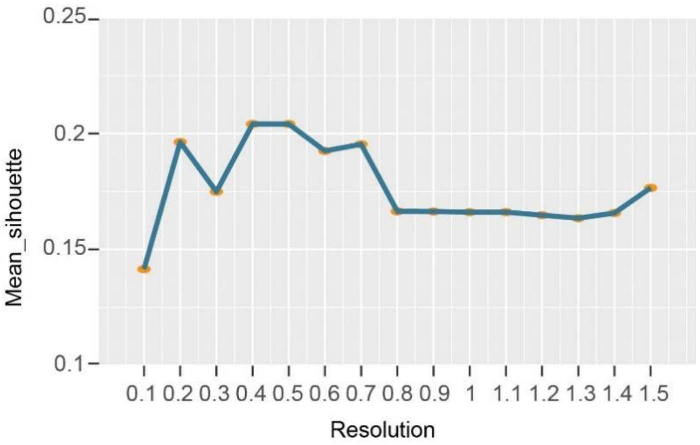

Figure S5. Plot of mean silhouette width at each resolution.

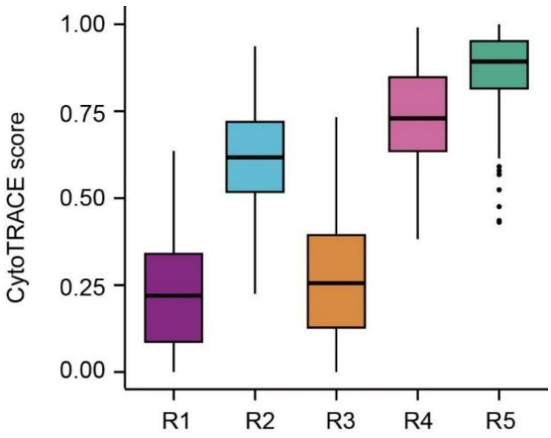

Figure S6. Boxplots of CytoTRACE score for five resistant sub-clusters.

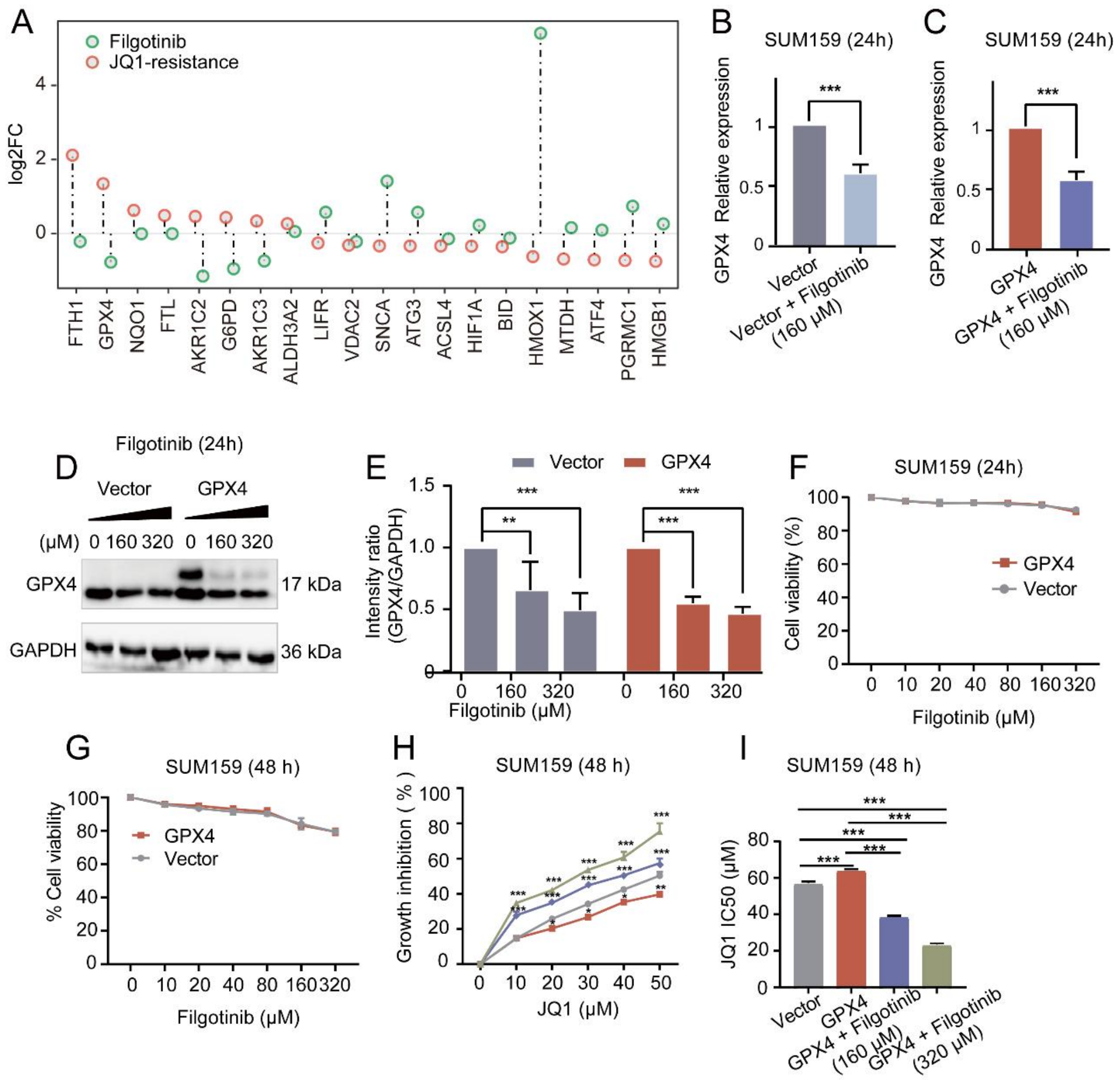

Figure S7. Cell viability experiments confirmed that filgotinib re-sensitizes TNBC cells to JQ1. **A**. Differential expression of 12 ferroptosis drivers and 8 ferroptosis suppressors in JQ1 resistance and fligotinib treatment, respectively. **B**. Relative mRNA expression of *GPX*4 in fligotinib (160 μM, 24h)-treated versus untreated Vector groups. **C**. Relative mRNA expression of *GPX*4 in fligotinib (160 μM, 24h)-treated versus untreated *GPX*4 overexpression groups. **D**. Western Blotting results of *GPX*4 in fligotinib (160 μM and 320 μM, 24h)-treated Vector and *GPX*4 overexpression groups. **E**. Western Blotting quantitative results of *GPX*4 in fligotinib (160 μM and 320 μM, 24h)-treated Vector and *GPX*4 overexpression groups. **F**-**G**. Cell viability of Vector and *GPX*4 overexpression SUM159 cell treated with filgotinib for 24 hours (**F**) or 48 hours (**G**). **H**-**I**. CCK8 assay of JQ1 inhibition rate (48h) in Vector and *GPX*4 overexpression groups (treated or untreated with fligotinib). Data represent mean ± SD, significance in (**B**-**C**) was calculated using the Unpaired-samples T tests (n=3). Significance in (**E**) and (**H**-**I**) was calculated using the one-way ANOVA (n=3). * $p < 0.05$; ** $p < 0.01$; *** $p < 0.001$.

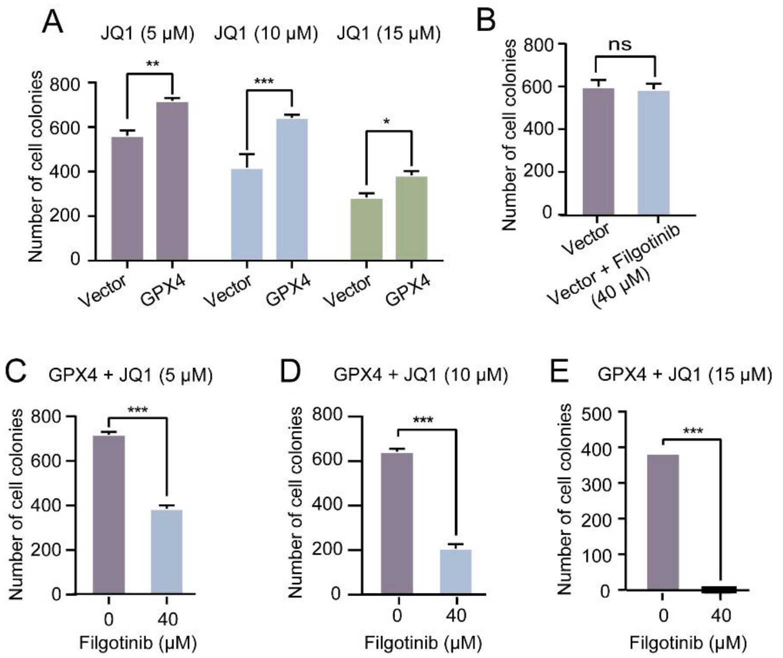

Figure S8. Colony formation assays confirmed that filgotinib re-sensitizes TNBC cells to JQ1. **A**. Quantification comparisons of the colony formation assays in *GPX*4 overexpression group vs Vector group (treated with JQ1 of 5, 10 and 15μM, respectively); **B**. Quantification comparisons of the colony formation assays in Vector group vs Vector treated with fligotinib (40μM). **C**-**E**. Quantification comparisons of the colony formation assays in *GPX*4 overexpression cells treated with JQ1 (5, 10 and 15μM, respectively) or JQ1-fligotinib (40μM) combination. Data represent mean ± SD. Significance in (**A**) was calculated using the one-way ANOVA (n=3). Significance in (**B**-**E**) was calculated using the Unpaired-samples T tests (n=3). * $p < 0.05$; ** $p < 0.01$; *** $p < 0.001$.

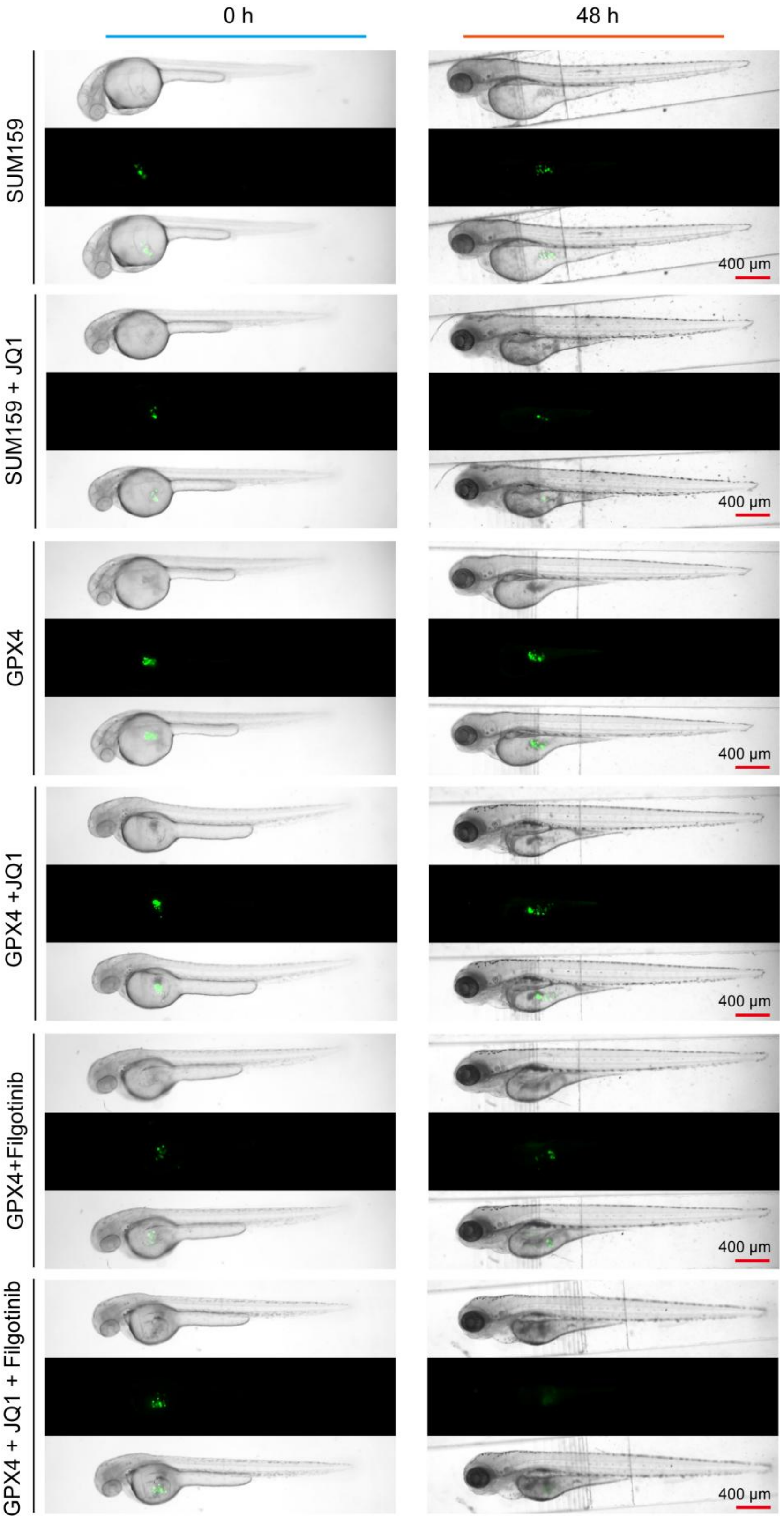

Figure S9. *In vivo* experiments confirmed that filgotinib re-sensitizes TNBC cells to JQ1. Representative images of xenograft zebrafish at 0 hours and 48 hours across different groups. Scale bar = 400 μm.

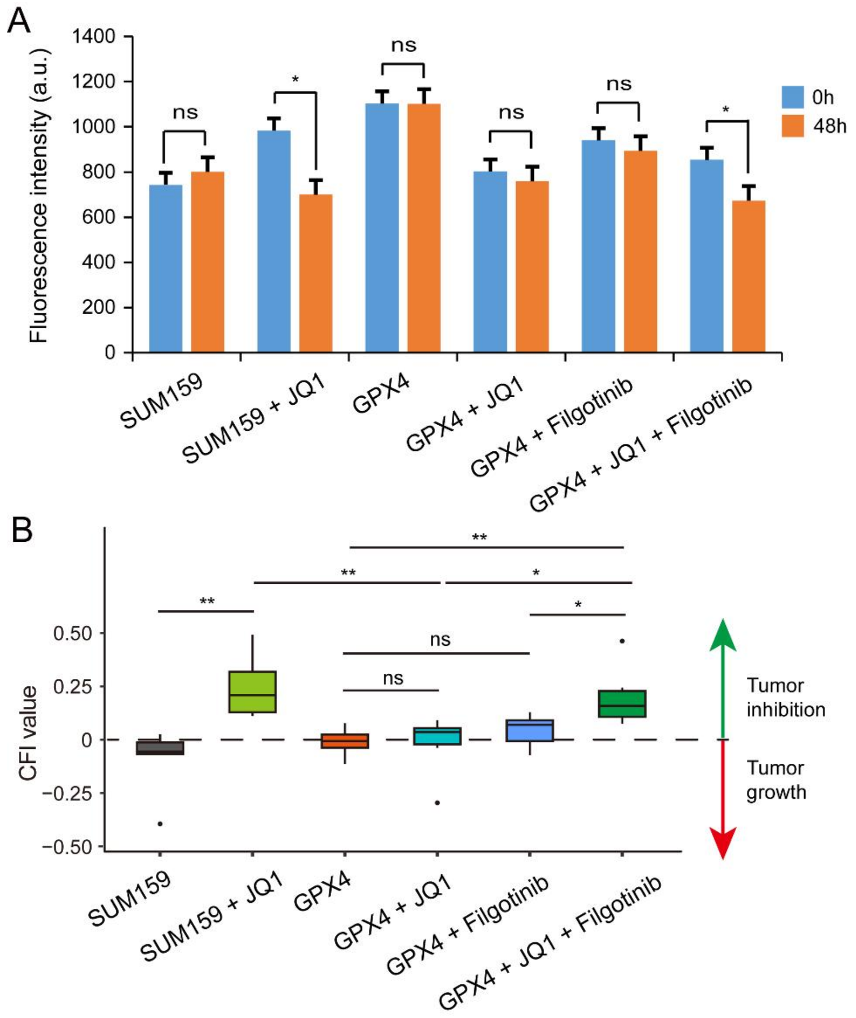

Figure S10. Quantitative results of xenograft zebrafish images (Figure S9). **A**. The bar graphs illustrate the differences in tumor cell fluorescence intensity between 0 and 48 hours. **B**. The box plots illustrate the differences in magnitude of change of fluorescence intensity (CFI) across different groups. Data represent mean ± SD in the bar graphs, significance in (**A**) was calculated using the paired-samples T tests (n=6). Significance in (**B**) was calculated using the Unpaired-samples T tests (n=6). The box plots show the median and the 25th and 75th percentiles. * $p < 0.05$; ** $p < 0.01$.

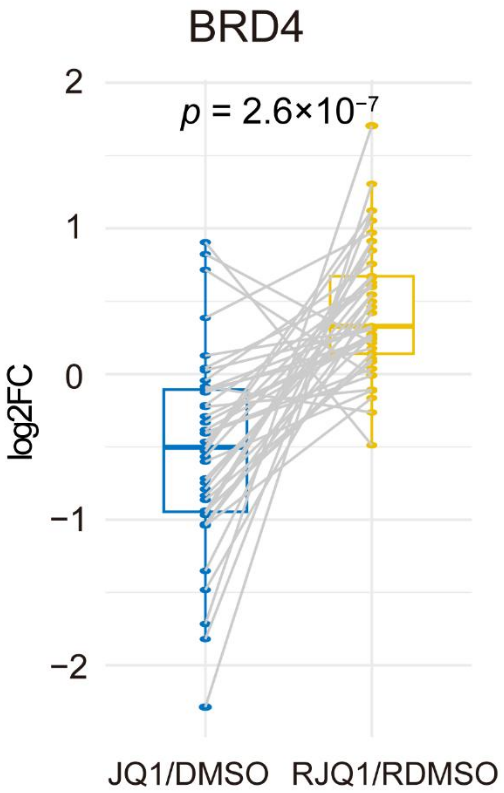

Figure S11. Changes in BRD4 binding affinity to ferroptosis-related genes across drug-response states. Box plots depict BRD4 ChIP-seq signal intensities at ferroptosis-related gene loci in SUM159 TNBC cells under four conditions: parental (DMSO, sensitive), JQ1-treated (JQ1) and JQ1 resistant (RDMSO), as well as JQ1 resistant cells retreated with JQ1 (RJQ1). Data were derived from the GEO dataset GSE131135. Statistical significance between groups was assessed using the two-sided Wilcoxon Rank-Sum test.

Uncropped Western blot membranes

Fig. S3C

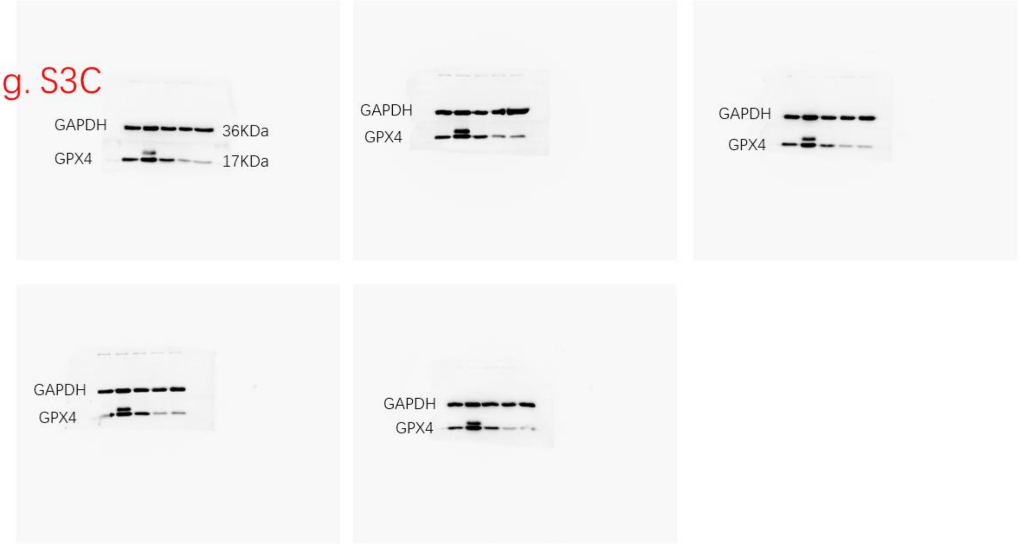

Fig. S7D

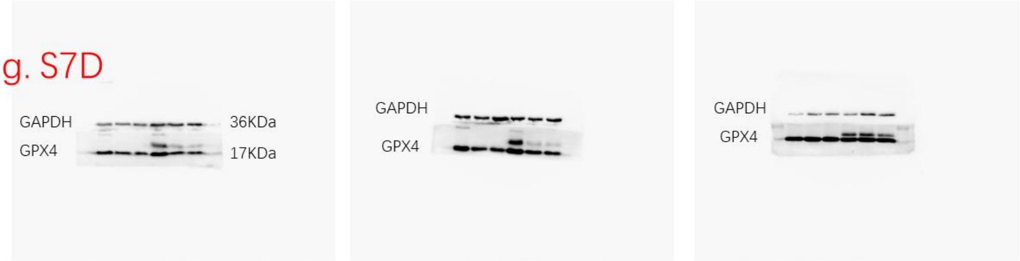

Supplementary Tables

Title: Supplementary Table 1.
Description: Top 20 markers ranked by avg_log2FC in each stage.

Title: Supplementary Table 2.
Description: Average expression of each gene in six dynamic patterns.

Title: Supplementary Table 3.
Description: KEGG pathways enriched with the DEGs between resistant and sensitive cells.

Title: Supplementary Table 4.
Description: Significant differential activity of transcription factors, which could regulate ferroptosis regulators, between resistant and sensitive cells.

Title: Supplementary Table 5.
Description: KEGG pathways significantly and specifically up-regulated in each resistant sub-cluster.

Title: Supplementary Table 6.
Description: Predicted top 10 JQ1 re-sensitizers by D-FR20.
